# Visual gene developer: a fully programmable bioinformatics software for synthetic gene optimization

**DOI:** 10.1186/1471-2105-12-340

**Published:** 2011-08-16

**Authors:** Sang-Kyu Jung, Karen McDonald

**Affiliations:** 1 Department of Chemical Engineering and Materials Science, University of California, Davis, 1 Shields Ave, Davis, CA 95616, USA

## Abstract

**Background:**

Direct gene synthesis is becoming more popular owing to decreases in gene synthesis pricing. Compared with using natural genes, gene synthesis provides a good opportunity to optimize gene sequence for specific applications. In order to facilitate gene optimization, we have developed a stand-alone software called Visual Gene Developer.

**Results:**

The software not only provides general functions for gene analysis and optimization along with an interactive user-friendly interface, but also includes unique features such as programming capability, dedicated mRNA secondary structure prediction, artificial neural network modeling, network & multi-threaded computing, and user-accessible programming modules. The software allows a user to analyze and optimize a sequence using main menu functions or specialized module windows. Alternatively, gene optimization can be initiated by designing a gene construct and configuring an optimization strategy. A user can choose several predefined or user-defined algorithms to design a complicated strategy. The software provides expandable functionality as platform software supporting module development using popular script languages such as VBScript and JScript in the software programming environment.

**Conclusion:**

Visual Gene Developer is useful for both researchers who want to quickly analyze and optimize genes, and those who are interested in developing and testing new algorithms in bioinformatics. The software is available for free download at *http://www.visualgenedeveloper.net*.

## Background

Several biotech companies provide gene synthesis services at an affordable price. Any DNA sequences can be designed and synthesized with a fast turnaround time of less than 1 month. At the present time, the bottom line price has already dropped to about $0.35/base for individual customers. So it currently costs only $500 (US Dollar) to synthesize a typical gene of 1.5 kilo base pairs with sequence confirmation. Although researchers relied on natural sources or collaborators to get genes in the past, direct synthesis of genes has become a more economic and reliable way to obtain source genes.

Moreover, it has a great advantage in that it allows the redesign and optimization of native DNA sequences to improve gene expression. There have been many successful reports demonstrating over-expression after sequence optimization. One of the most popular approaches is codon optimization for heterologous gene expression. The idea is that host species have different preferred codon usages for amino acid incorporation and unfavorable codons in a foreign gene can be replaced with favorable codons while maintaining the expression of the same amino acids. Although it is still controversial and recent work has called into question its usefulness as a predictor of expression in some hosts, CAI (Codon adaptation index) or codon bias has been one of the most commonly used indexes to evaluate genes. In a rational manner, codon optimized genes that imitate a codon bias of especially highly expressed genes or exclude rare codons could significantly increase gene expression level [1-6]. According to results from recent large scale experiments, gene expression level varied more than 40 fold in 40 variants and up to 250 folds in 154 variants [7,8]. These studies provide compelling evidence that synthetic gene design can have a significant impact.

However, the mechanism of gene expression is complicated at the molecular level and codon bias is not the only determinant for gene expression efficiency. For example, there is an active debate on the relationship between codon bias, mRNA folding energy, and gene expression. Welch et al. synthesized more than 40 variants of two genes each and compared actual expression levels in *E. coli *[7]. They identified the most important 10 codons encoding a subset of amino acids that strongly accounted for expression level. However, they couldn't find a correlation between mRNA folding energy and expression probably due to weak mRNA binding energy across the synthetic gene library. Tuller et al. have a slightly different finding from a genome scale study [9]. They concluded that although the gene expression level of individual genes can't be determined simply from a correlation between folding energy and expression level, mRNA folding energy still strongly modulates translation efficiency which is correlated with codon bias determined by tRNA adaptation index. Moreover, Kudla et al. developed a larger collection of 154 synthetic genes coding for GFP and tried to find a rule for gene expression [8]. Their results showed that mRNA folding energy at sites near the ribosomal binding sites had a dominant effect on gene expression for efficient translation initiation and also that codon bias measured by CAI wasn't associated with GFP expression level. Therefore, the issue of the influence of codon bias and mRNA folding energy on protein production is currently open to discussion. Moreover, regarding gene design criteria, there are many other factors such as Shine-Dalgarno or Kozak's context sequence, repeated sequences, potential polyadenylation sites, cryptic splice sites, introns, and nuclease cleavage sites as well as restriction enzyme sites, GC content, UTR (untranslated region), and use of rare codons that affect gene expression.

Due to the complicated gene design criteria, gene optimization is not easy since it requires huge repetitive computations. Thus, it is highly necessary to use software design tools to assist in the process of gene design, analysis, and optimization. There are several software packages currently available such as Codon optimizer [10], DNAWorks [11], DyNAVacS [12], GeMS [13], Gene Composer [14], Gene Designer [15], GeneDesign [16], GeneOptimizer [17], JCat [18], OPTIMIZER [19], Synthetic Gene Designer [20], and UpGene [21]. However, one of the challenges arising in the implementation of gene optimization algorithms is that the "ideal" gene optimization algorithm has not been fully established since we still have very limited understanding of gene expression and regulation mechanisms. Furthermore, the development of algorithms will require extensive coupling, perhaps in an iterative fashion, between experimental studies and computational model development. Currently, high level expression and/or accurate prediction of heterologous synthetic gene expression cannot be guaranteed, particularly in diverse host organisms. Therefore, there is a need to develop flexible software that offers a greater variability of gene optimization strategies as well as modeling tools, and the ability to couple experimental studies with algorithm development. At the same time, the software needs to provide diverse options and toolboxes for gene analysis to help researchers with the study of synthetic gene design.

**Figure 1:**
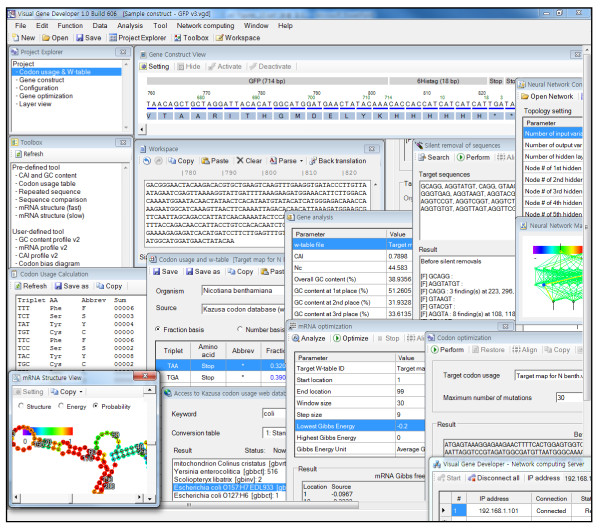
**Screenshot of Visual Gene Developer showing basic functions**. Current version carries about more than 30 user-friendly GUI windows that provide specialized functions for gene analysis and optimization.

In order to facilitate gene design, we have developed a unique gene design software called Visual Gene Developer (Figure 1). The software provides a user-friendly interface and includes many useful functions such as mRNA secondary structure/binding energy prediction, codon usage/mRNA optimization, GC content/Nc (effective number of codons)/CAI calculation, sequence comparison, repeated sequence search, multiple query sequence search, and silent removal of undesirable sequences. Visual Gene Developer's interface has been optimized to handle a large number of genes and can perform batch analysis for a genome-scale study. In particular, the function to predict mRNA secondary structure or optimize the stability of mRNA is important for optimization since mRNA structure or Gibbs free energy may affect translation initiation and elongation and consequently protein synthesis. A recent review paper provides a good summary on the issue [22]. Furthermore, the software not only supports network and multi-threaded computing that reduce the processing time significantly but also includes an artificial neural network prediction toolbox, a nonlinear multivariable method that has not been implemented in other software. The artificial neural network regression toolbox may be potentially useful to analyze and optimize sequences since some researchers have used artificial neural network models to identify gene expression patterns [23], signal peptides [24], intron splice sites [25,26], and translation initiation sites [27], and others have demonstrated the usefulness of linear empirical modeling tools such as the PLS (Partial least square) prediction toolbox to identify important codons and predict gene expression level [7]. One of the powerful features that only Visual Gene Developer has, is that the software supports script language programming using Visual basic script (VBScript) and Java script (or JScript), and allows a user to develop new algorithms in an integrated programming environment where built-in editing/test tools, 80 helper functions of gene analysis and optimization, and a customizable graphics interface are provided. Therefore, the software allows researchers to test new optimization strategies or gene analysis metrics possibly allowing the discovery of novel rules in terms of gene expression with minimal programming effort. As a result Visual Gene Developer enables more flexibility and expandability compared with other commercial or free software that is currently available.

## Implementation

**Figure 2:**
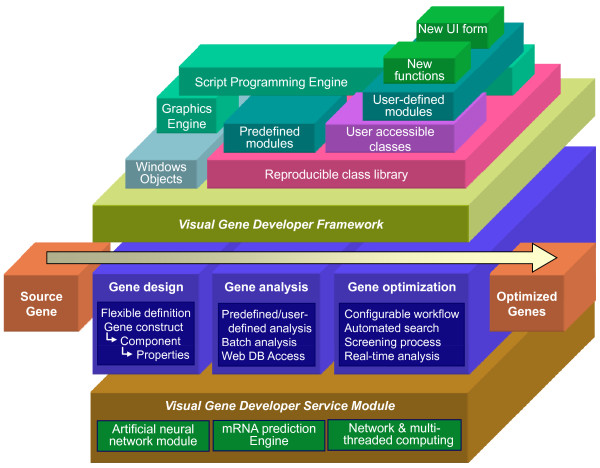
**Software architecture**. 'Visual Gene Developer Framework' allows a user to utilize implemented functions and develop new modules, and Service Modules were developed to provide 3 useful tools. Any gene can be quickly designed, analyzed, and optimized in the integrated software environment.

As a Microsoft Windows application, Visual Gene Developer has been developed using Microsoft Visual Studio 2010 and built on .Net™(dot Net) Framework. Like other Windows software, Visual Gene Developer contains interactive windows for gene design, analysis and optimization, and has an intuitive interface for the user. The software consists of several functional modules as shown in Figure 2 and most algorithms were compacted into classes. For simplicity, a class can be defined as a reproducible programming object that contains a collection of functions. In order to provide a programming environment like Mathematica™ or Matlab™, a script programming engine was developed along with a module editor and in the current version (1.0 Build 619) there are 9 essential classes (AppService, GeneService, GeneConstruct, mRNApredict, NeuralNet, NetComService, PropertyBag, CustomUI, and ScriptService) available for module development. Each class contains many useful functions. For instance, the 'GeneService' class contains more than 60 helper functions to assist with sequence manipulation and analysis. In addition, several specialized routines are also available to access GUI (Graphical User Interface) objects of Visual Gene Developer. For example, a user can use the function 'Workspace_Value' of the class 'AppService' to read (or write) a sequence from (to) a text box of the Workspace window and utilize graphics functions of the class 'CustomUI' to generate user-defined GUI windows to show analysis results as a figure. Meanwhile, three service modules such as artificial neural network, mRNA secondary structure prediction, and Network and multi-threaded computing are implemented that can be used either as classes, user-friendly windows or toolboxes.

### Interface

The software consists of a main menu bar, a toolbar, and other module windows with a graphical user interface. The main menu bar that is located at the top of the screen contains all features and functions. As a collection of sub menu items, the 'Project Explorer' window provides quick links to other module windows in connection with the target codon usage table, the gene construct, and configuration for gene optimization strategy or parameters. The 'Gene construct view' window shows a graphical representation of a gene construct including DNA/amino acid sequence, gene construct component name, and size or location of bases. The 'Toolbox' window has a configurable menu system where user-defined functions selectively can be added as menu items. Double-clicking on one of menu items, a user can execute its algorithm. The 'Workspace' window is a place where a sequence is analyzed and manipulated. Many menu functions will work for the sequence in the 'Workspace' window. For module development, there are four essential windows such as 'Module Library', 'Module Editor', 'Property Bag Library', and 'Property Bag Editor' windows. Regarding the data file format, Visual Gene Developer saves, exports, and imports all data files as a standard ASCII text format that is easily accessible by other editing software. We disclosed the data file structure and allow anyone to make use of generated data files including Project, Script, Property Bag, codon usage table, and neural network files. Furthermore, for convenient data exchanges between Visual Gene Developer and other software such as Microsoft Excel™, SigmaPlot™, or Origin™, we provide copy and paste function at many locations of the software.

#### (1) Gene design, analysis, and optimization

As one of the most important features, Visual Gene Developer not only contains many useful functions to facilitate gene design and analysis but also provides versatile optimization strategies. Since most basic functions are integrated into the main menu of the software, a user can easily manipulate and analyze a sequence. For example, the 'Function' section of the main menu includes parsing, translating, reversing a sequence, and generating complimentary sequence as well as silent removal of undesirable sequences and codon/mRNA optimization for a sequence in the 'Workspace' window. The 'Analysis' section of the menu covers calculating codon usage, CAI, GC content, Nc, searching multiple query sequence or repeated sequence, comparing two sequences, and predicting mRNA secondary structure. To build a codon usage table for CAI calculation or codon usage adaptation, the software has a function to directly import codon usage tables from CUTG (Codon Usage Tabulated from GenBank) website (*http://www.kazusa.or.jp/codon/) *and calculate *RSCU *(Relative Synonymous Codon Usage), *RSCU_max_*, and *w *value (Relative adaptiveness of a codon), and also helps users build their own local codon usage database for highly expressed genes within genomes under translational selection. Regarding sequence optimization, although a user can simply use basic functions mentioned above, the software provides a more advanced tool to solve complicated optimization problems. Using 'Gene Construct Designer' window, a user can design a gene construct that has several different gene construct components, configure optimization conditions in the 'Configuration of Gene Optimization' window, and run the optimization process in the ''Gene Optimization' window. The optimization system is based on the combination of multiple algorithms. A user can select several different algorithms to design a unique optimization strategy. For example, the simplest combination may be using 'Monte-Carlo Codon Optimization' and 'Check CAI' functions. The former function is to optimize a gene and the latter is to screen out an improper gene when its CAI value is lower than a setpoint. The implemented algorithms include performing silent removal, checking repeated sequences, restriction enzyme sites, Gibbs free energy of mRNA secondary structures, and etc.

#### (2) Programming capability

As already mentioned, there have been numerous trials to improve heterologous gene expression by modifying the DNA sequence, including codon optimization. However, it is still not clear what the best gene design criteria is because of insufficient knowledge of gene expression and regulation mechanisms at the molecular level, particularly for a wide variety of potential host organisms. Therefore, currently there exists no generally accepted way to optimize gene designs and many researchers are trying to develop and improve gene optimization algorithms. Of course each algorithm needs to be confirmed by experiments.

**Figure 3:**
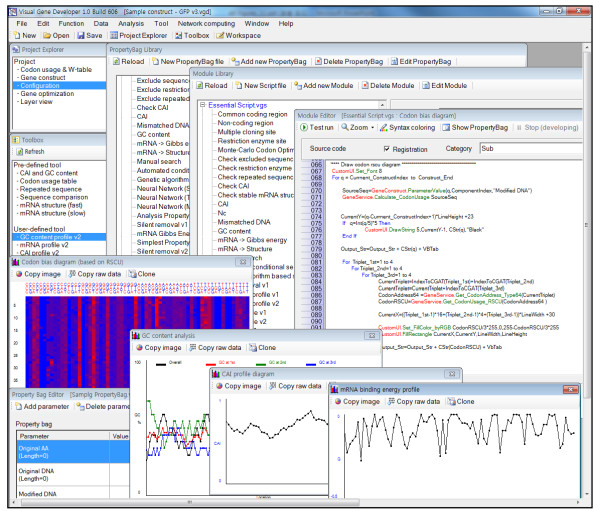
**Integrated programming environment for module and custom UI development**. For module development, 4 specialized windows such as 'Module Library', 'Module Editor', 'PropertyBag Library', and 'PropertyBag Editor' are provided (top and left bottom). 4 CustomUI forms (bottom) titled 'Codon bias diagram', 'GC content analysis', 'CAI profile diagram', and 'mRNA binding energy profile' are shown to demonstrate how user-defined modules work.

In order to provide a very flexible way to design a new algorithm, Visual Gene Developer supports script language programming and allows users to develop new functions and plug-ins to expand the functionality of the software (Figure 3). For example, although several useful gene analysis index parameters such as CAI, GC content, mRNA secondary Gibbs free energy, and Nc are known and also available in Visual Gene Developer, there are still many opportunities to identify other useful indices. To test a new finding, a user can manipulate algorithm and easily add the function to the software. To offer programming capability, the software has an integrated script engine and interface to process two popular script languages: VBScript (Visual Basic script) and JScript (Java script). A user can develop and test a new module in the 'Module Editor' window and manage modules in the 'Module Library' window to delete or edit modules. To facilitate code development, Visual Gene Developer has a unique UI called a configurable Property Bag that can contain several different properties. As a window UI object where an end-user can set parameter values, a Property Bag can be defined by the user and associated with the module. Using a specialized class, 'PropertyBag', a module developer can read or write a value of a property calling property name. In that way, a Property Bag works as a visual communication port between an end-user and a module developer to change the behavior of a module even though an end-user may not recognize user-defined algorithm and interface. Two module windows ('PropertyBag Editor' and 'PropertyBag Library') are used to define Property Bags. A user can deploy a module to a specified location of a window according to defined module category.

As platform software, Visual Gene Developer provides more than 170 helper functions that are compacted into classes. Essential classes and their functions are summarized in Table 1.

**Table 1:** Summary of user-accessible classes and functions

Class name	Subject	Example of functions
AppService	Miscellaneous application service (6 functions)	- Export data to clipboard - Access to Workspace window - Show instant message

GeneService	Sequence manipulation, analysis, optimization (62 functions)	- Parse, translate, reverse, etc. - Calculate CAI, Nc, GC contents - Search query/repeated sequences - Do codon/mRNA optimization - Remove undesired sequences silently

GeneConstruct	Access Gene construct and components (12 functions)	- Manipulate gene construct components - Get current gene construct index - Access to a real time analysis table

mRNApredict	Prediction of mRNA secondary structure (18 functions)	- Predict mRNA structure and binding energy - Visualize predicted structure - Read raw data of mRNA structure

NeuralNet	Artificial neural network prediction module (6 functions)	- Open trained network map file - Set input variables - Predict outputs

CustomUI	Custom GUI form and graphics engine (27 functions)	- Draw line, circle, rectangles, pie, arc, and text - Export raw data and image

NetComService	Network and multi-threaded computing module (23 functions)	- Connect/disconnect service - Get information about server, client, jobs

PropertyBag	Manipulation of Property Bag (11 functions)	- Clear and add property - Read/write parameter values

ScriptService	Execution of user defined module (6 functions)	- Load/run user-defined script module - Assign parameter array - Retrieve results

#### (3) mRNA secondary structure prediction

Visual Gene Developer carries a sophisticated mRNA prediction engine that can be used to optimize a gene construct by adjusting mRNA binding energy or structure. The engine was developed as a class by embedding the stand-alone mRNA prediction software called Vienna RNA Secondary Structure Package developed by Hofacker [28]. For module developers, Visual Gene Developer provides a user accessible class named as 'mRNApredict'. The class contains many simplified functions that can be used with a single line code in Visual Gene Developer's programming environment to predict MFE (minimum free energy) mRNA secondary structure, calculate mRNA binding energy, visualize a predicted structure, or read raw data of the predicted structure. Therefore, it is highly useful to develop more complicated optimization algorithm modules that utilize mRNA structure prediction. For example, local Gibbs free energy of mRNA structure can be repeatedly calculated to generate a binding energy profile over a test sequence.

Visual Gene Developer also includes simple menu functions and a specialized window form for mRNA structure prediction. After putting a sequence into a textbox in the 'Workspace' window or 'mRNA structure' window and clicking on menu or 'Calculation' button, a predicted mRNA secondary structure will be quickly shown in the 'mRNA structure viewer' window with a graphical representation of base pairing, probability of base pairing, energy, and locations. The 'mRNA structure viewer' window also has a function to export the image or raw data to a clipboard.

With regard to mRNA structure optimization, a common problem is that it usually requires a large number of repeated calculations of mRNA binding energy. Definitely, it takes a much longer time to find optimal mRNA structure compared with codon optimization. To reduce the overall calculation time, the mRNA prediction engine was designed and optimized for network and multi-threaded computing as will be explained later. Utilizing several computers or multiple threads in a computer, multiple processes of mRNA structure prediction can be performed at the same time to analyze multiple gene constructs or gene construct components. Thus Visual Gene Developer can be used to analyze mRNA secondary structures of several thousand genes for genome-scale studies as well as gene optimization in itself.

#### (4) Artificial neural network module

An artificial neural network model can be defined as a non-linear computational model that consists of a number of highly interconnected artificial neurons to simulate the structure and function of biological neural networks. The goal is mapping a set of input patterns onto a corresponding set of output patterns. It has been widely used to model complicated nonlinear systems that consist of multiple variables to predict data patterns.

Due to the potential application of artificial neural network models for gene analysis and optimization, Visual Gene Developer includes an artificial neural network prediction toolbox. The software has a configuration window to design the topology and adjust learning parameters and can directly import (or export) a data set from the clipboard or typical ASCII text format files. A typical feedforward neural network with a standard back propagation learning algorithm to train networks was implemented [29].

As with other classes, Visual Gene Developer allows a user to programmatically access the neural network toolbox and class. This means that users can utilize artificial neural network models when they develop new modules. For example, it is possible to predict gene expression levels of gene constructs in real time during the gene optimization process if a user has already developed and trained neural networks to correlate several different parameters to the expression level. In addition, the software contains useful functions to analyze the trained neural network map and test input and output variables as 2-D or ternary diagrams.

#### (5) Network & multi-threaded computing

Since gene analysis and optimization processes usually require extensive computations, it may take long time to find optimal genes if there are a lot of genes to analyze and/or if the gene analysis algorithms are complicated. For example, in the case of mRNA secondary structure prediction, it takes about 30 seconds to calculate a global mRNA secondary structure for a typical size gene of 1~1.5 kilo base pairs and it will take at least 3 days to generate mRNA secondary structures of 10,000 genes using a Pentium 4 computer. In order to reduce the calculation time, a grid/parallel computing using a workstation or a supercomputer is very useful [30-32]. However, the problem is that it is not easy for non-experts to access to those computing resources.

Network or multi-threaded computing can be a good alternative and Visual Gene Developer supports this approach for the calculation of mRNA structure or binding energy as well as user-defined functions. The term 'network computing system' generally refers a system that utilizes multiple computers that are connected to a network to perform computational tasks. The idea is that if several computers are available and they are connected to the internet, Visual Gene Developer connects those computers and splits the server's work load to the connected computers. For example, in order to calculate mRNA binding energy of 10 genes, Visual Gene Developer's server transfer gene sequence data to 10 client computers and then receives calculated results from them. Thus the theoretical processing time can be reduced to 1/10 of the original processing time if data transfer time is neglected. On the contrary to network computing, multi-threaded computing means a utilization of multiple threads in a single core or a multi-core computer. The concept of multiple threads or multiple processes has a close relationship with multi-tasking in a computer. For reference, a computer or an operating system runs an application on a process and each process consists of at least one thread. As an actual programming code, a thread performs a certain job. However, in spite of great advantages of multi-tasking, it significantly reduces overall performance of a single application. To overcome this intrinsic problem, Visual Gene Developer executes multiple client processes on a single computer and links them together on a local network. In this way, Visual Gene Developer simulates a multi-threaded computing system that makes use of all computer resources more efficiently.

Visual Gene Developer has a robust network & multi-threaded computing module with a user-friendly interface and provides a specialized class that contains several useful functions to monitor network connections. Because all functions including data communication protocol were implemented into a single executable file, the software doesn't require any expensive hardware and has an ability to operate both a server and multiple clients at the same time. Once computers are connected to the internet, Visual Gene Developer allows a user to easily setup a server and clients in the 'Server' window. On the client side, a user can add new clients by choosing 'Add client' in the main menu. The 'Job list' window shows the current status and includes functions to pause the service or cancel reserved jobs.

## Results

### Gene design

Visual Gene Developer has a hierarchical and expandable system to define a gene construct and gene construct components. A gene construct is defined as a full length sequence that consists of several gene construct components as building blocks. Each gene construct component has a collection of predefined properties that is referred to as gene construct component type. A property which has its own name and storage space works as a variable that can be used to store information such as DNA sequence. A gene construct component type determines the data structure of a gene construct component, and each gene construct component can possess several different sequence or non-sequence data.

As an example, a gene construct may have 4 different gene construct components such as 5' HindIII restriction enzyme site, Shine-Dalgarno sequence, GFP coding sequence, and 3' multiple cloning site in consecutive order. Each gene construct component belongs to one of the predefined gene construct component types such as 'Coding sequence', 'Non-coding sequence', 'Restriction enzyme site', or 'Multiple cloning site'. In case of 'Coding sequence', it has 3 different properties whose names are 'Original AA', 'Original DNA', and 'Modified DNA' where AA stands for amino acid. Each property holds amino acid sequence or DNA sequence information. In contrast to 'Coding sequence', 'Non-coding sequence' like Shine-Dalgarno sequence doesn't need amino acid sequence data. Therefore, the gene construct component type of 'Coding sequence' has only two properties: 'Original DNA' and 'Modified DNA'.

For reference, the 'Original DNA' sequence data can be used only for special purposes such as to calculate mismatched bases or codons between the original and variant sequences whereas 'Modified DNA' is inevitably used as an essential property that most modules utilize to modify, optimize, analyze, read, and write. With regard to sequence or data size, the software permits both variable length and null size (zero length) of a sequence. Therefore a user can modify the sequence length during the gene optimization process and even generate an "invisible" gene construct component that will not be shown in the 'Gene Construct View' window by setting the 'Modified DNA' to be null.

Furthermore, the software lets a user define new gene construct component types to hold unique information and functions. A gene construct component type can be easily designed in the 'PropertyBag Editor' and 'Module Editor' windows. However, to be functional, it is usually necessary to develop new modules or modify existing modules to handle new properties.

As a main part of the user interface, the software has the 'Gene Construct Designer' window. A user can design a gene construct by adding or deleting gene construct components, or changing a location of a gene construct component. When adding a new gene construct component to a gene construct, a user can choose a gene construct component type among the defined gene construct component types that are listed in a drop down list box in the window.

### Sequence analysis

Visual Gene Developer includes basic functions to manipulate a sequence such as sequence parsing, back translation, and conversion into the reverse sequence or the complementary sequence. After putting the source sequence into a text box in the 'Workspace' window, a user can click on one of the main menus to perform the function. Otherwise, a user can develop a module to modify the input sequence in the 'Workspace' window and then expose the module on the 'Toolbox' window as a typical menu item. For gene analysis purposes, the software includes several gene analysis algorithms for calculating the CAI, GC content, and Nc, Codon usage table, and performing sequence comparisons, repeated sequence searches, multiple sequence searches, and mRNA secondary structure prediction. The software also supports a batch processing to analyze several thousands of genes. After setup in the 'Gene Optimization' and 'Gene Construct Designer', a user can import a ASCII text file that contains multiple sequence data and then check the analysis result in the 'Gene Optimization' window. Owing to the programming capability, a user can make use of implemented classes and add new gene analysis metrics or predictions to Visual Gene Developer.

#### (1) CAI (Codon adaptation index)

The CAI has been widely used as an effective measure of synonymous codon usage bias. It was originally proposed by Sharp and Li to quantify the extent of codon usage similarity between a reference set of genes and a gene of interest [33]. The CAI ranges from 0 to 1 where higher CAI means highly codon biased or higher codon usage similarity between two different codon usage tables. In order to calculate the CAI, we follow the same procedure and make use of the original definition given by Sharp and Li [33]. For reference, the software calculates the *RSCU *(Relative Synonymous Codon Usage) from a codon usage table of a reference gene and then computes *w_i _*(Relative adaptiveness of a codon) value for each codon by dividing *RSCU *by *RSCU_max_*. Finally, CAI value can be calculated using the following equation.

CAI= exp1L⋅∑i=118∑j=1kiXij lnwij

where *X_ij _*is the total number of the *j*th codon for the *i*th amino acid in the test gene, *w_ij _*is the relative adaptiveness of the *j*th codon for the *i*th amino acid in the reference gene, *k_i _*is the number of synonymous codons for the *i*th amino acid, and *L *is the total number of codons excluding AUG (Met) and UGG (Trp) in the test gene. As a special case, if *w_ij _*is smaller than 0.01, it is adjusted to 0.01 [34].

#### (2) Nc (Effective number of codons)

This quantity was originally defined by Wright [35] to measure a degree of codon bias. It is a number between 20 and 61 where 20 means extremely biased and 61 stands for equally biased between synonymous codons. In contrast to CAI, the calculation of Nc doesn't need a reference codon usage table. First of all, the software calculates codon homogygosity ($\hat{F}$) of the amino acid [35].

$${\hat{F}}_{i}=\frac{\left(n\overset{k_{i}}{\underset{j=1}{\sum }}p_{j}^{2}\right)-1}{n-1}$$

where ${\hat{F}}_{i}$ the codon homogygosity of the *i*th amino acid, *n *is the total number of the amino acid in the test gene, *k_i _*is the total number of synonymous codons of the *i*th amino acid, and *p_j _*is the codon frequency of the *j*th allele (synonymous codon).

The effective number of codons is then calculated by summation of the average homogygosities.

$${\hat{N}}_{c}=2+\frac{9}{{\bar{\hat{F}}}_{2}}+\frac{1}{{\bar{\hat{F}}}_{3}}+\frac{5}{{\bar{\hat{F}}}_{4}}+\frac{3}{{\bar{\hat{F}}}_{6}}$$

where ${\bar{\hat{F}}}_{m}$ (*m *= 2, 3, 4, or 6) is the average homogygosity for the amino acids whose total number of synonymous codons is *m*. For example, ${\bar{\hat{F}}}_{6}=\left({\hat{F}}_{Arg}+{\hat{F}}_{Leu}+{\hat{F}}_{Ser}\right)∕3$.

As Wright suggested, if some amino acids are missing then Visual Gene Developer computes the average homogygosity by taking an average of homogygosities of amino acids present in the test gene. If isoleucine is absent or rarely used, Fuglsang's estimation is used to calculate ${\bar{\hat{F}}}_{3}$[36].

$${\bar{\hat{F}}}_{3}={\hat{F}}_{Ile}=\frac{{\left(\frac{2}{{\bar{\hat{F}}}_{2}}-1\right)}^{-1}+{\left(\frac{2}{3{\bar{\hat{F}}}_{4}}+\frac{1}{3}\right)}^{-1}+{\left(\frac{2}{5{\bar{\hat{F}}}_{6}}+\frac{3}{5}\right)}^{-1}}{3}$$

#### (3) Repeated sequence search

The software allows a user to identify repeated sequences in a test sequence. It detects not only forward directional and backward complimentary repeated sequences but also palindromic sequences and consecutively connected repeated sequences. In order to find repeated sequences, a moving window method was employed. The algorithm generates a short sequence clipped from a test sequence and then compares the partial sequence with the test sequence to find matched locations. The search process is repeated while the moving window scans along the test sequence. When the scanning is completed, potentially duplicate findings are removed if they are already included in other findings. The feature is named as the 'Smart filter' in the 'Search sequences' window.

#### (4) Multiple query sequence search

This function was developed to identify locations of query sequences within a sequence. A user can input a set of multiple query sequences or restriction enzyme names separated by Tab, comma (,), or Carriage Return (Enter key) in the 'Search sequences' window. For in-depth analysis a query string is split into multiple strings of single query sequence. In case of restriction enzyme names, they are converted into DNA sequences. After performing repeated searches for all query sequences in a test sequence, the software shows detailed information about the total number of occurrences and their locations in a gene for every query sequence. A user can choose one of a predefined sequence set such as common restriction enzyme sites, potential intron cryptic splice sites or polyadenylation signal sequences.

#### (5) Profile calculation of CAI, mRNA Gibbs free energy, or GC content

The software contains 3 implemented modules that are used to calculate a profile of CAI, mRNA binding energy, or GC content of a test sequence. Their algorithms are quite similar between them as the moving window approach was equally adopted and their codes were developed from the same template code. In general, any single calculation such as GC content can be repeatedly performed while a moving window is sliding over a test sequence. The procedure is initiated when a moving window is located at the first base of the test sequence. For example, mRNA binding energy of the first 60 bases is calculated if the size of the moving window is set to be 60 bases that can be adjusted by the user. After the first calculation, the moving window steps forward to the next location such as to the 11th base of the test sequence if the step size of the moving window is 10 bases. In this way, the RNA binding energy is repeatedly computed at an interval of 10 bases until the moving window arrives at the end of the sequence. To generate data for a profile plot, both the location of the moving window and its corresponding mRNA binding energy are recorded as a table format. Since the codes were written in VBScript, a user can easily modify source codes to develop new profiling functions.

### Sequence optimization

Visual Gene Developer contains useful modules to optimize a gene construct in terms of codon usage, mRNA binding energy, known conserved sequence, or undesirable sequence. Owing to programming capability, a user can develop new modules utilizing simplified helper functions of the classes mentioned earlier.

#### (1) Codon optimization

The software provides a predefined module that is based on a well-known Monte-Carlo simulation or a predefined probability table [13,15,19,20]. It utilizes a codon usage table and replaces original codons with new ones while maintaining the identity of the same amino acids. To be specific, Visual Gene Developer not only has a function to import codon usage tables from CUTG (Codon Usage Tabulated from GenBank) but also provides a manual edit mode for the target codon usage map and allows a user to generate a local database of reference sets of optimal codon usage tables. The software automatically calculates the *RSCU, RSCU_max_*, and *w_i _*values, and then generates a look-up table (LUT) of synonymous codons. For example, if alanine has four synonymous codons such as GCA, GCC, GCG, and GCT whose expected fractions are 0.1, 0.2, 0.3, and 0.4, respectively, the LUT will consist of 100 GCAs from 1 to 100, 200 GCCs from 201 to 300, 300 GCGs from 301 to 600, and 400 GCTs from 601 to 1000 in a memory array. Finally, one of 1000 codons is randomly chosen and then it replaces the original codon. By utilizing the look-up table, it is possible to perform codon optimization very quickly. In addition, the software has a pre-defined function that allows a user to keep track of changes in codon usage bias as a graphical representation. Meanwhile, since the current version of the software doesn't have a built-in database of optimal codon usage maps of highly expressed genes, a user may need to rely on other available sources including papers and web databases where a user can get an optimal codon usage map for a specific host genome and then put it into Visual Gene Developer.

#### (2) mRNA optimization

In order to optimize a gene in terms of mRNA binding energy, the algorithm was developed utilizing both mRNA prediction and codon optimization modules. At the code level, an original sequence is continuously modified until its Gibbs free energy is in a specific range given by minimum or maximum Gibbs free energy where the modification refers to synonymous substitution. With regard to the modification strategy, the simplest approach is that the number of mutations is gradually increased one by one when the calculated Gibbs free energy is out of range. Meanwhile, the base module was also used to develop more complicated modules. For example, a binding energy profile of a long test sequence can be optimized by repeatedly applying the base algorithm to all local sequences with a moving window method. In this way, all local mRNA structures can be optimized while minimizing the number of base changes. Similarly, a user can increase or decrease binding energy at specific locations in a sequence. Visual Gene Developer has a specialized window for mRNA optimization for a typical user and provides related class functions for a module developer.

#### (3) Removal of undesirable sequences

**Figure 4:**
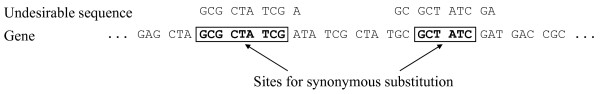
**Schematic diagram of algorithm for silent removal**. Target sequences are identified, truncated and then replaced with synonymous codons.

The coding region of a gene may include undesirable sequences such as restriction enzyme sites, potential polyadenylation signal sequences, or cryptic splice sites. Visual Gene Developer provides a function to remove such unwanted sequences without changing the resulting amino acid sequence. The algorithm is based on the synonymous substitution and similar to that for codon optimization except it replaces only a few codons with correspondent synonymous codons in the target sequence region that needs to be modified (Figure 4). The first step is to identify the location of the target DNA sequence in a gene and then determine the location of the site for synonymous substitution. To simplify the substitution process, terminal sequences of the target sequence are truncated if they are located outside of complete codons. The current version of the software carries 4 relevant modules that are written in VBScript. A user can easily remove undesirable sequences including predefined potential polyadenylation signals and intron cryptic splice sequences.

### Optimization process

To help users design unique optimization processes, Visual Gene Developer has a versatile and configurable optimization strategy and interface. First of all, the optimization process is based on a novel combination of multiple modules. Each independent and fully functional module does a simple job like codon optimization or silent removal. By integrating individual modules into a comprehensive optimization process, it is possible to implement a more complicated and diverse gene optimization strategy. A user can easily add new modules by choosing one of the listed algorithms in the 'Configuration for Gene Optimization' window. At the same time, a user can determine their priority or the order of module execution. For module development, there are 5 different types of optimization modules such as 'Sequence optimization', 'mRNA structure optimization', 'Gene manipulation', 'Constraint' and 'Search strategy'. Especially, 'Search strategy' belongs to a global optimization module that determines the optimization process and controls all other modules.

Secondly, the software has an ability to generate and handle a large quantity of candidate gene constructs that satisfy the user's gene design criteria. This is important because the number of possible variant genes is practically infinite even after codon, mRNA structure, or UTR optimization in spite of screening out of undesired gene constructs. Simply, the number of possible variants of a gene can be calculated to be $3.28^{\frac{18}{20}n}$ if we assume equal probability between 20 amino acids where *n *means total number of amino acids of the gene and 3.28 is an average number of synonymous codons of 18 amino acids that have multiple codons. For instance, if a gene consists of 250 amino acids, total number of possible variants is about 1.18 × 10^116^. One cycle of the optimization process will generate single candidate gene constructs and multiple cycles will produce many candidate gene constructs. A user can check all generated gene constructs in the 'Gene Optimization' window. As one interesting feature of the software, a generated gene construct can have its origin like a relationship between mother and daughter, and a user can specify a source gene construct for the next round of the optimization process. The option is useful to find desirable sequences step by step in a short time.

Thirdly, the software has a built-in screening system to remove undesirable gene constructs. A researcher may prefer using active processes such as silent removal of unwanted sequences. However, the screening process can be a faster and simple way to find good candidate gene constructs. Any designed modules that are registered as 'Constraint' type will be used to determine whether a gene construct satisfies a certain set of criteria or not. If a module returns a 'Not pass' value, the current gene construct will be discarded.

#### (1) Excluding query sequences or specified restriction enzyme sites

The purpose of the module is to avoid undesirable DNA sequences including potential cryptic splice sites, polyadenylation signal, and restriction enzyme sites. When those sequences are found, the gene construct will be excluded from the candidate gene construct list.

#### (2) Checking stability of mRNA secondary structure

This algorithm is a modification of the 'mRNA Gibbs free energy plot'. It is used to analyze mRNA secondary structures of all partial sequences of a sequence. If the calculated Gibbs free energy of a local sequence in a moving window is lower than a threshold value, the module returns 'Not pass' value and consequently the gene construct will be screened out.

#### (3) Removing repeated sequences

In order to prevent repeated sequences in a gene construct, the module is developed to count the total number of repeated sequences in a sequence. If the number is more than a prescribed set point, the gene construct will be ruled out.

### Comparison with other similar web servers and software

Basically, most available software including Visual Gene Developer share a similar codon optimization strategy. Monte Carlo algorithm or 'one amino acid-one codon method' is frequently adopted [19]. For high gene expression, several programs such as Gene Composer, Gene Designer, JCat, OPTIMIZER, and Synthetic Gene Designer include optimal codon usage maps of highly expressed genes. Regarding mRNA secondary structure optimization, Gene Composer and Visual Gene Developer carry the most sophisticated modules. Both software have functions to eliminate stable mRNA hairpin structure and control Gibbs free energy utilizing advanced mRNA folding algorithms. GeneOptimizer, Gene Designer, GeMS, and JCat don't calculate Gibbs free energy of mRNA folding. However, they indirectly eliminate potential mRNA structure sequences by analyzing sequence repetitions or calculating energy scoring functions in a short range of a test sequence. The other software tools such as Codon optimizer, DNAWorks, DyNAVacS, GeneDesign, OPTIMIZER, Synthetic Gene Designer, and UpGene don't have a function to predict mRNA secondary structure and don't perform mRNA optimization that means the use of Gibbs free energy analysis to assess the stability of mRNA secondary structure. Compared with other available software, Visual Gene Developer has several novel implementations that have not been implemented elsewhere such as artificial neural network modeling, integrated programming environment using VBScript and JScript (= Java script), network/multi-threaded computing, and sophisticated batch analysis and optimization process for multiple gene construct candidates. However, one of the limitations is that Visual Gene Developer is platform-dependant as a Microsoft Windows™ application whereas other software supports multiple platforms or web browsers (DyNAVacs, GeneDesign, Gene Designer, GeneOptimizer, JCat, OPTIMIZER, Synthetic Gene Designer). In addition, further development is needed to include other useful features such as a built-in database of codon usage tables of highly expressed genes or robust regression toolboxes like PLS (partial least square) or SVM (support vector machine) model that have not been implemented yet.

## Conclusion

As an emerging research area, synthetic gene design studies have demonstrated that well designed gene constructs can greatly improve gene expression level. Not to mention that good software is definitely an essential tool to optimize a gene or discover new gene design optimization criteria. Visual Gene Developer offers a variety of built-in features as well as the ability for the user to incorporate new functions. The software's framework is well designed and the built-in design concepts or features are good examples that can inspire the development and incorporation of new modules by other workers, including unique optimization algorithms for complicated non-linear, multivariable systems.

## Availability and requirements

Visual Gene Developer is available for free download at *http://www.visualgenedeveloper.net*. The website provides a full description of the software and functions, and includes helpful tutorials covering common gene design approaches to custom module development. The software supports Windows XP/Vista/7 and requires at least an Intel Pentium 3-class processor or equivalent working at 800 MHz and 512 MB of RAM.

## Authors' contributions

SKJ conceived and developed the software. SKJ also wrote the manuscript and built the software website. KM initiated the project and provided feedback on the software development and manuscript. Both authors read and approved the manuscript.
